# Differences between Pygmy and Non-Pygmy Hunting in Congo Basin Forests

**DOI:** 10.1371/journal.pone.0161703

**Published:** 2016-09-02

**Authors:** Julia E. Fa, Jesús Olivero, Miguel Angel Farfán, Jerome Lewis, Hirokazu Yasuoka, Andrew Noss, Shiho Hattori, Masaaki Hirai, Towa O. W. Kamgaing, Giuseppe Carpaneto, Francesco Germi, Ana Luz Márquez, Jesús Duarte, Romain Duda, Sandrine Gallois, Michael Riddell, Robert Nasi

**Affiliations:** 1 Division of Biology and Conservation Ecology, School of Science and the Environment, Manchester Metropolitan University, Manchester, M1 5GD, United Kingdom; 2 Center for International Forestry Research (CIFOR), CIFOR Headquarters, Bogor, 16115, Indonesia; 3 Grupo de Biogeografía, Diversidad y Conservación, Departamento de Biología Animal, Universidad de Málaga, Facultad de Ciencias, Campus de Teatinos s/n, 29071, Málaga, Spain; 4 Department of Anthropology, University College London, 14 Taviton Street, London, WC1H 0BW, United Kingdom; 5 Center for African Area Studies, Kyoto University, 46 Yoshida-Shimoadachi-cho, Sakyo-ku, Kyoto, 606–8501, Japan; 6 Department of Geography, University of Florida, 3141 Turlington Hall, Gainesville, FL, 32611–7315, United States of America; 7 Faculty of International Studies, Tenri University, 1050 Somanouchi, Tenri City, Nara, 632–8510, Japan; 8 Japan Forest Technology Association, 7 Rokubancho, Chiyoda-Ku, Tokyo, 102–0085, Japan; 9 Ministry of Forestry and Wildlife, P.O. Box 14794, Yaoundé, Cameroon; 10 Dipartimento di Scienze, Università Roma Tre, Rome, Italy; 11 Ofitecma, calle Colombia 5, 29400, Ronda, Spain; 12 Ethnoecology Laboratory, Universitat Autònoma de Barcelona, 08193, Bellaterra, Barcelona, Spain; 13 Département Hommes, Natures, Sociétés MNHN—Musée de l'Homme 17 place du Trocadéro, 75116, Paris, France; 14 Bioclimate, Research and Development, UN House, 4 Hunter Square, Edinburgh, EH1 1QW, United Kingdom; 15 Consultative Group on International Agricultural Research (CGIAR), CIFOR Headquarters, Jalan CIFOR, Situ Gede, Bogor, 16115, Indonesia; Sichuan University, CHINA

## Abstract

We use data on game harvest from 60 Pygmy and non-Pygmy settlements in the Congo Basin forests to examine whether hunting patterns and prey profiles differ between the two hunter groups. For each group, we calculate hunted animal numbers and biomass available per inhabitant, P, per year (harvest rates) and killed per hunter, H, per year (extraction rates). We assess the impact of hunting of both hunter groups from estimates of numbers and biomass of prey species killed per square kilometre, and by examining the proportion of hunted taxa of low, medium and high population growth rates as a measure of their vulnerability to overhunting. We then map harvested biomass (kg^-1^P^-1^Yr^-1^) of bushmeat by Pygmies and non-Pygmies throughout the Congo Basin. Hunting patterns differ between Pygmies and non-Pygmies; Pygmies take larger and different prey and non-Pygmies sell more for profit. We show that non-Pygmies have a potentially more severe impact on prey populations than Pygmies. This is because non-Pygmies hunt a wider range of species, and twice as many animals are taken per square kilometre. Moreover, in non-Pygmy settlements there was a larger proportion of game taken of low population growth rate. Our harvest map shows that the non-Pygmy population may be responsible for 27 times more animals harvested than the Pygmy population. Such differences indicate that the intense competition that may arise from the more widespread commercial hunting by non-Pygmies is a far more important constraint and source of conflict than are protected areas.

## Introduction

Modern humans have occupied and used the Congo Basin forests for at least 50,000 years. Evidence of Pygmy culture dates back more than 20,000 years. Pygmies are the largest group of nomadic or semi-nomadic indigenous hunter–gatherers in sub-Saharan Africa, found exclusively within the main forest blocks in the Congo Basin [1,2]. Today, these traditional hunter-gatherers have complex, multi-generational relationships with farmers, exchanging forest products for starch-rich foods and access to manufactured goods. In contrast, some 29 million non-indigenous people, comprising more than 150 distinct Bantu and non-Bantu peoples [3], overlap with Pygmies. For both Pygmies and non-Pygmies, bushmeat, along with fish, is a traditional food staple and a significant protein source. However, the importance of wild meat in the diets of these forest dwellers varies considerably depending on modes of procurement but also on the availability of supply. The latter is dependent on the structure and composition of the forest ecosystems themselves as well as on the intensity, duration and periodicity of timber and non-timber resource extraction, including hunting.

The conservation of biodiversity within tropical forest areas whilst taking into account the needs of human communities is still much debated. The dispute essentially falls around whether conservation projects and policies should prioritize biodiversity and landscape protection, or poverty alleviation and sustainable human livelihood improvement. Ultimately, the underlying issue focuses on the real or potential impacts that people have on biodiversity and the degree to which benefits and costs of conservation should be shared [4–7]. In its simplest form, the debate has separated those who advocate people-free or ‘fortress conservation’ [8] and those in favor of people-centered conservation [9]. Nonetheless, conservation today encompasses a spectrum of approaches, which vary in the degree to which they balance objectives of biodiversity conservation with those emphasizing human livelihoods [7, 10].

Those aiming at the protection of tropical forest biodiversity have historically followed two general approaches, one aimed at safeguarding wild species and natural systems (by establishing parks and other protected areas), and the other by promoting restraint in the harvest and consumption of wild species and their products [11]. This latter approach can occur under a variety of participatory forest management mechanisms such as forest co-management, community forestry, and other forms of Community Based Natural Resource Management (CBNRM) such as those customary forest management regimes recognized by National Governments.

Both approaches affect people’s access to natural resources, either by denying them the opportunity to use certain areas (as in protected areas), or by reducing their harvest levels. In so doing, conservation actions can conflict with other ethical obligations, by curtailing, for instance, the ability of some people to make a living, an obligation and a core right recognized in the Universal Declaration of Human Rights [12]: ‘‘Everyone has the right to a standard of living adequate for [their] health and wellbeing”.

Often, the goals of animal conservation and the needs of indigenous peoples wishing to use wildlife as a food resource, bushmeat, are not always compatible. This lack of concordance in goals is particularly urgent given the increasing debate over the conflict between protected areas in the Congo Basin and the needs of local people [13,14]. Though analyses of resource use conflicts between forest peoples and protected area managers in specific localities are informative, basin-wide analyses of wildlife extraction levels by indigenous and non-indigenous groups can allow us to better understand existing and future incompatibilities between subsistence and large-scale commercial hunting. Commercial hunting is an economic activity involving hunting as a way of life, final purchasers or consumers, and often a chain of middlemen. Such levels of hunting have increased significantly due to rapid human population growth, socioeconomic change, infrastructure development and technological improvements [15]. A wide variety of terrestrial vertebrates are consumed as bushmeat, with ungulates, rodents and primates constituting the majority [16]. Estimates for bushmeat harvested across the Congo Basin range from 1 million t Yr^–1^ [17] to over 4.5 million t Yr^–1^ [18, 19]. Such levels of harvesting are deemed unsustainable; estimates suggest wildlife extraction is occurring at more than 6 times the sustainable rate [20, 21].

In this paper, we use a comprehensive compilation of hunting studies quantifying terrestrial vertebrate kills brought into Pygmy and non-Pygmy settlements in the Congo Basin. We first explain the differences between hunter groups in the composition of wild species hunted and provide evidence that non-Pygmy hunters differ significantly from Pygmy hunters. We then quantify extraction levels per unit area of the two hunter groups and model the distribution of hunting pressure throughout the Congo Basin region. Finally, we discuss the extent to which hunting by Pygmies and non-Pygmies can be defined as sustainable, and how the conservation objectives of the protection of biodiversity in Central Africa can be made compatible with the needs of indigenous and non-indigenous peoples.

## Methods

### Study Site Selection Criteria

Data on animal kill records for Pygmy and non-Pygmy hunters were compiled from the literature, and unpublished studies. The following selection criteria were used in amassing these studies: (1) tropical forest within the distribution range of Pygmies (according to Olivero et al. [22]) was the predominant vegetation type within the hunting catchment area; (2) the number of individuals and identification of all species brought to the settlement surveyed were available; and (3) settlement size (number of inhabitants in the settlement at the time of the study) and the number of hunters involved in the study was available.

### Study Settlements

**Fig 1:**
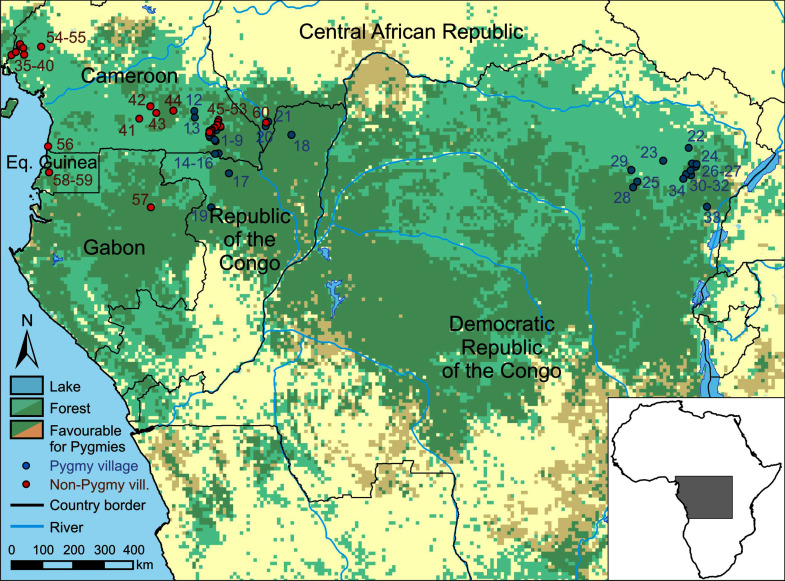
Location of Pygmy and Non-Pygmy sites considered in this study. Locality numbers refer to game harvest profiles listed in S1 Table.

We compiled game harvest data from 34 hunting studies involving Pygmy camps and other settlements and another 26 studies on hunting by non-Pygmies (Fig 1; S1 Table). The Pygmy sample encompassed studies representing Baka/Aka/Efe groups from Cameroon (n = 16), Republic of Congo (n = 3), Central African Republic (n = 2) and Democratic Republic of Congo (n = 13). Non-Pygmy studies included non-Pygmy groups from Cameroon (n = 21), Republic of Congo (n = 1), Central African Republic (n = 1), Gabon (n = 1) and Equatorial Guinea (n = 2).

Although ecoregions varied across sites, the predominant vegetation in all study sites was Lower Guineo-Congolian forest. This type of forest extends from the coast of the Atlantic Ocean in the west to the mountains of the Albertine Rift in the east and spanning nearly 7 degrees north and south of the equator [23].

### Game Harvest Profiles

From each study, we documented the number of individual animals of reptiles, birds and mammals hunted in Pygmy and non-Pygmy settlements (S2 Table). We excluded from the analyses those taxonomic entries with uncertain definitions (e.g. ‘small squirrels’). For all species, we adopted nomenclature in the IUCN Red List [24]. Common and scientific names of all recorded species are also given in S2 Table.

We also extracted information on the duration of each study, during which hunting data was logged, as well as the number of hunters responsible for the hunts. We also recorded proportion of hunted prey sold. We use the latter as a measure of dependence on bushmeat within the studied settlement, where higher proportions of bushmeat sold may suggest a greater emphasis on the commercialization of hunting to supply larger markets, including towns and cities [19].

For each species, we used the mean body weight available for male and female adults of each taxon from the literature in order to standardize comparisons between sites. This was necessary because weights of individual carcasses (or the aggregate biomass of all dressed or undressed carcasses of a given species) were not provided in several studies. Body mass data were extracted from Kingdon et al. [25] for all mammalian orders, from Dunning [26] for birds, and from L. Luiselli (pers. comm.) for reptiles.

Using body mass data and number of individual animals hunted, we estimated the mean body mass of mammals harvested within each study site. We focus on mammals because this class of vertebrates is by far the most important for both hunter groups in this study. We used the mean body mass as a proxy of species composition; a drop from larger to smaller species may indicate a process of defaunation of a habitat [27].

### Estimation of Harvest and Extraction Rates

Hunting methods will affect extraction levels since traditional capture devices such as bows, crossbows and nets result in different selectivities that cannot be easily compared with kills obtained from modern weapons such as cable snares and firearms. Although there were records of the variety of hunting methods used by the different groups, we did not have sufficient data to determine the influence of these on prey selection. Despite this caveat, we argue here that because Pygmies and non-Pygmies diverge considerably in their hunting practices, the latter concentrating more on snare hunting and use of firearms [16], our comparison of game profiles of the two hunter groups allows us to contrast the broad patterns of game harvest between them.

By employing those studies that specified: 1) the type and number of prey taken, 2) the length of time during which the data were collected and 3) the number of people linked to the hunting recorded, we calculated a harvest rate, similar to Redford and Robinson [28], for each game species:

$$Harvest\;rate=\frac{no.\;of\;animals\;killed}{no.\;of\;potential\;consumers\;x\;duration}$$ (1)

in which number of potential consumers includes both hunters and non-hunters, and duration of the study is measured in years. This index measures per capita yield (kg P^-1^ Yr^-1^) of game animals for the average person in a community in one year.

To calculate extraction rates per hunter, we divided the number of animals recorded in each study by the number of hunters in the settlement and by the number of study days. We also computed extraction rates per species per unit area (km^-2^) by first calculating the territory size for each settlement from the relationship between territory size (km^-2^) and size of human settlements, as indicated in Hoare [29]. Settlement size and territory size are significantly and positively correlated (*y* = 0.6945*x* + 1.1409; *R*² = 0.44, *p* = 0.00, n = 32).

Animal biomass extracted per hunter or per unit area was determined for each study site by using the product of the body mass for every hunted species (S2 Table) and the calculated harvest and extraction rates.

Because of the relatively small number of days (3–10 days) sampled in adjacent Ituri Pygmy settlements (Sites 22–34), we summed the numbers and biomass of prey taken in these sites to obtain an overall harvest and extraction rate. In Sites 45–53, number of days sampled per settlement was also low (7–12). However, for these cases, we used the reported sample days as done in Bobo et al [30].

### Impact on Species

As a measure of impact on the more threatened species, we recorded the number of species hunted by each hunter group that appeared as Critically Endangered (CR), Endangered (E), or Vulnerable (V) in the IUCN Red List [24].

We assessed the possible impact of Pygmy and non-Pygmy hunters on hunted mammal species from the harvest rates derived for each settlement. We determined the proportional number of individuals of all species hunted in each settlement according to each species’ fecundity rate. We used the calculated intrinsic rate of increase (*r*_max_) from Fa et al. [31] to categorize each species into low (0–0.25), medium (0.25–0.50) and high fecundity (>0.50) classes.

We assessed the relation between mean body mass of hunted mammals and the average human population density around each site to assess whether larger prey corresponded with less disturbed areas and whether these differences were related to hunter group. We used human population density as a proxy of anthropogenic pressure within a 20-km radius around each site, as in Fa et al. [32]. Human population density data was obtained from the LandScan™ 2008 High Resolution (1 km^2^) Global Population Data Set (copyrighted by UT-Battelle, LLC, operator of Oak Ridge National Laboratory).

### Mapping Potential Bushmeat Consumption

Bushmeat consumption was mapped by combining georeferenced information on human population with our estimations of average harvest rate (kg) per person using Eq 1 Human population/km^-2^ with respect to Pygmy groups was estimated according to the environmental favourability model for Pygmy occurrence proposed by Olivero et al. [22]. We calculated the Pygmy potential population size (PPS) for every grid cell of the study area (0.1° × 0.1°), according to favourability values in Olivero et al. [22]. From this, the Pygmy population density was computed using the following equation, taking territoriality into account:

$$Population\;density=\frac{PPS\;x\;GCS}{GCS}=\frac{PPS}{ATS}$$ (2)

where GCS is the size of a grid cell, and ATS is the average territory size estimated for Pygmy settlements (i.e. 1,079 km^2^, see Olivero et al. [22]).

For non-Pygmies, we estimated the rural population density by combining two data sources: (1) human population density, calculated utilizing the LandScan™ 2008 High Resolution (1 km^2^) Global Population Data Set (copyrighted by UT-Battelle, LLC, operator of Oak Ridge National Laboratory); and (2) urban areas, taken from MODIS 500-m Map of Global Urban Extent, produced using data circa 2001–2002 [33, 34]. We then calculated average population density values within 0.1° × 0.1° grid cells. Bushmeat consumption per grid cell was finally calculated by multiplying population density by the average harvest rate per person as estimated above. Pygmy and non-Pygmy populations were treated separately.

Total potential consumption of bushmeat in Central African forests by Pygmy and non-Pygmy populations was computed by multiplying the resulting harvest rates for each hunter group by the surface-area of every grid. We then summed the resulting values in all grids within the forest area, defined by forest classes (1 to 5) of the MODIS Collection 5 Global Land Cover Map [35]. All spatial analyses and representations were performed using the software ArcGIS 10.3.

### Statistical Analyses

All means are given with their associate standard deviations (SD). We employed one sample t-tests to compare means between hunter groups.

## Results

### General

Pygmy settlements (villages along logging roads as well as familial forest camps) examined here had on average 91.9 inhabitants (S.E. = 24.3, range = 8–690, n = 33. Non-Pygmy settlements, all villages, were larger (882.4 ± 311.0 inhabitants, range = 35–6594, n = 22). Average length of studies was 66.2 ± 13.7 days (range = 3–368 days) for Pygmies, and longer (279.6 ± 54.4 days, range = 7–1020 days) for non-Pygmies. Percentage number of hunters in Pygmy settlements was 32.2 ± 3.7% (range = 0–79.1%, n = 31), and lower in non-Pygmy ones (24.3 ± 6.5%, range = 0.6–100%, n = 31).

Sale of hunted game varied significantly between the two hunter groups (*p* = 0.03); on average 34.8 ± 6.4% (range = 0–90%, n = 26) of the hunted game in Pygmy settlements was sold, whereas significantly more prey (65.4 ± 19.8%, range = 11–95.3%, n = 24) was commercialized in non-Pygmy settlements. For both hunter groups, there was no correlation between the proportion of animals sold and human population density (*p* = 0.50). In those Pygmy sites where no trade was recorded (n = 11) human population density varied from a minimum of 0.9 inh. km^-2^ to 140.8 inh. km^-2^. However, there was no difference between mean human population densities of sites where no bushmeat was traded and those sites where prey were sold (*p* = 0.08).

Only Pygmy hunters used traditional hunting methods (bows and arrows, crossbows, nets, spears, vine traps), documented in 67% of the Pygmy studies considered here. Spear hunting was recorded in 49% of the settlements, net hunting in 33%, use of traditional traps in 33%, and bow and arrow in 30%. Cross bow use was documented in only one study. Cable snares were, however, common to 64% of the settlements but shotgun hunting was only documented in 15% of the sites. Use of machetes or capture by hand of less mobile prey (e.g. tortoises, python and pangolins) was typical in 18% of the settlements. Non-Pygmy hunters used only cable snares and shotguns.

We recorded a total of 859 hunters active in Pygmy settlements, who were responsible for 4,069 animals killed in 2,250 study days–a total of 3,450,512 hunter-days. By contrast, our survey included less non-Pygmy hunters (n = 660), who together killed 52,611 animals in 6,812 study days; 4,380,245 hunter-days.

### Game Harvest Profiles

Pygmies and non-Pygmies hunted a total of 123 species in our study; 1 amphibian (0.80%), 11 reptiles (8.9%), 21 birds (17.1%) and 90 mammals (73.2%). Out of these, 48 species were common to both groups; 2 birds (4.44%), 39 mammals (86.67%) and 4 reptiles (8.9%). Species could not always be compared directly because hunter groups were found in distinct faunistic regions of the Congo Basin. Hence, some harvested species occurred only in the eastern sites and others exclusively in western sites, e.g. the Weyn’s duiker *Cephalophus weynsi*, and the closely related Peter’s duiker *Cephalophus callipygus*, respectively. Equally, there were species limited to Cameroon, Equatorial Guinea, CAR and northern Congo that were not distributed in the east. For instance, some primates were typical of the far western forest region (e.g. black colobus *Colobus satanas*, drill *Mandrillus leucophaeus*, moustached guenon *Cercopithecus cephus*, greater white-nosed monkey *Cercopithecus nictitans*), while other species are endemic of the far eastern forest region (e.g. l'Hoest's Monkey *Cercopithecus lhoesti*).

**Fig 2:**
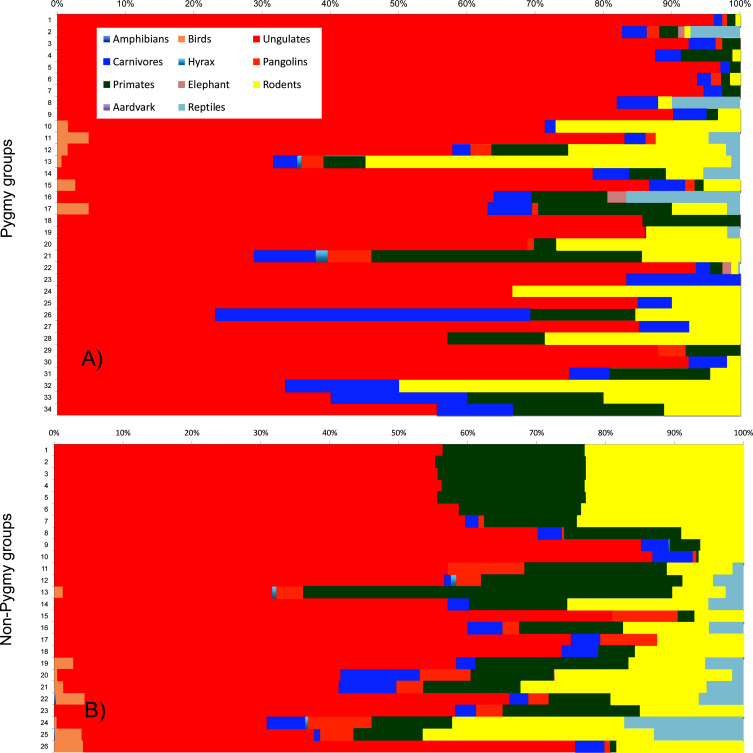
Stacked bar graphs, with sites sorted according to the numbering in Fig 1, of the percentage contribution made by the different taxonomic animal groups hunted by Pygmies and non-Pygmies.

The contribution made by the different animal groups varied significantly between hunter groups (Fig 2). Pygmy hunters killed a total of 77 species of terrestrial vertebrates; 62 mammals (74.2%), 8 birds and 7 reptiles. Non-Pygmy hunters took more species; 97 in total, of which 71 were mammals, 17 birds, 8 reptiles and 1 amphibians. Diversity of hunted species, as indicated by the Shannon index, was higher for non-Pygmies (2.1 ± 0.1; range 0.9–2. 5) than for Pygmies (1.7 ± 0.1; range 1.6–2.8). Species dominance values for hunted species were also higher for non-Pygmies (8.8 ± 0.6, range 4.9–16.1) than for Pygmies (6.0 ± 0.6, range = 2.5–11.6).

Among mammals, ungulates were the most hunted group by Pygmies (76.1%) and non-Pygmies (53.7%). Significant differences between both hunter groups also were observed in the percentage of rodents (Pygmies– 13.7%; non-Pygmies– 22.0%), primates (Pygmies– 4.3%; non-Pygmies– 16.7%) and carnivores (Pygmies– 2.7%; non-Pygmies– 3.3%) hunted.

**Fig 3:**
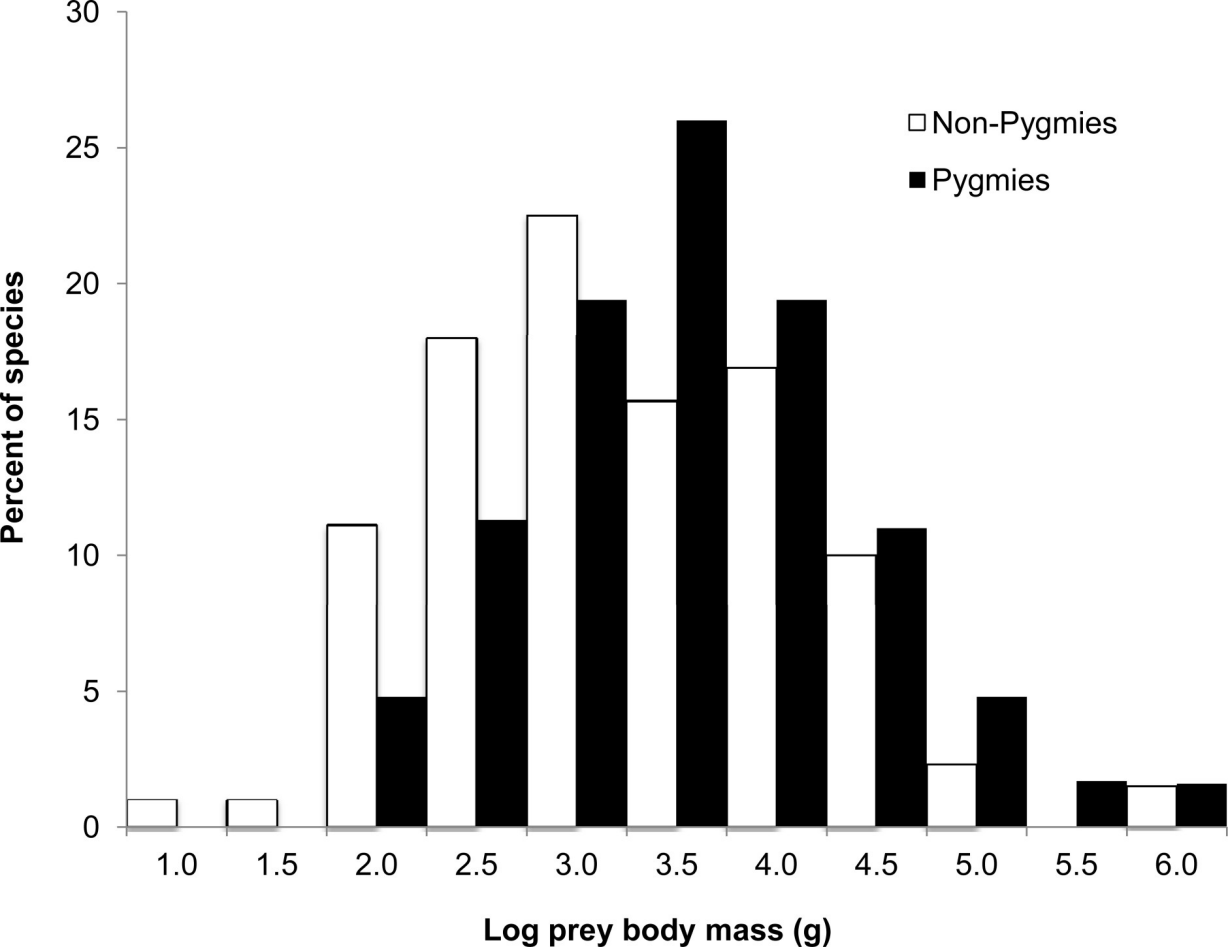
Size distribution of forest vertebrate kills harvested by Pygmies (n = 34 studies) and Non-Pygmies (n = 26), in terms of the (log_10_-transformed) body mass (in grams) of all recorded whole carcasses (adults and juveniles) of animal species (Pygmies = 71 spp.; Non-Pygmies = 122 spp.) hunted by each hunter group.

Prey items ranged widely in both hunter groups, from Zenker's pygmy anomalure *Anomalurus zenkeri* (17 g) to forest elephant *Loxodonta cyclotis* (1742 kg) in the case of Pygmies, and from the 20g crested chameleon *Trioceros cristatus* to the forest elephant for non-Pygmies. As shown in the overall distribution of prey species body mass (Fig 3), potential prey items smaller than 1 kg were rarely taken by Pygmy hunters, who concentrated primarily on larger mammals.

**Fig 4:**
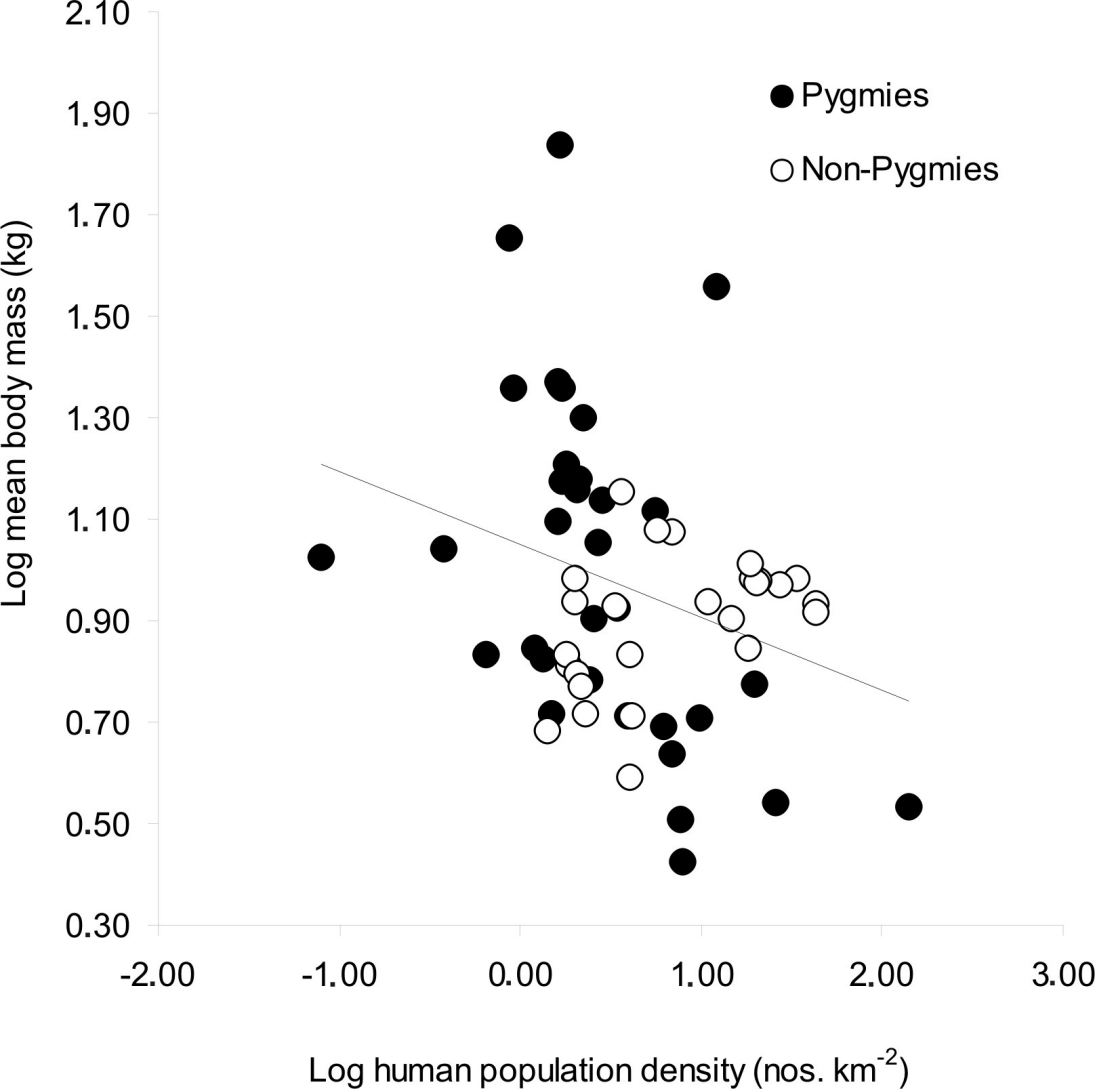
Relationship between mean body mass of mammals hunted in Pygmy and non-Pygmy sites and human population density in 20-km buffers around each site. Human population density is used here as a proxy for anthropogenic pressures in each study site.

For mammal species alone, the mean body mass of species hunted by Pygmies was significantly larger (14.2 ± 13.5 kg) than for non-Pygmies (8.3 ± 2.4 kg). The between-site variation in mean body mass was larger in Pygmy sites (range = 2.7–68.6 kg) than in non-Pygmy sites (range = 3. 9–14.2 kg). For both hunter groups (Fig 4), mean body mass was negatively correlated with human population density (*R* = -0.26, *p* = 0.05). However, the trend was steeper for Pygmy sites (*R* = -0.40, *p* = 0.02) but positive in non-Pygmy sites (*R* = 0.41, *p* = 0.04).

### Harvest and Extraction Rates

Per capita harvest rates calculated separately for all Pygmy and non-Pygmy sites (Table 1) showed that there were almost twice as many hunted animals per inhabitant in non-Pygmy sites but no significant difference in prey biomass per inhabitant was found between hunter groups. Likewise, the number of animals taken per hunter in non-Pygmy settlements was also significantly higher than in Pygmy sites. However, in biomass terms, Pygmy hunters took as much as non-Pygmy ones.

**Table 1:** Per capita harvest and extraction rates for Pygmy and non-Pygmy settlements.

	Harvest rates^a^		Extraction rates^b^	
	(P^-1^ Yr^-1^)		(H^-1^ Yr^-1^)	
	Prey numbers	Biomass (kg)	Prey numbers	Biomass (kg)
**Pygmies**				
N sites	23	22	22	21
Mean (SD)	20.4 (23.2)	376.3 (515.1)	87.9 (109.9)	1646.6 (2095.7)
Min	0.1	1.5	0.9	24.5
Max	76.8	1740.6	404.6	8183.7
**Non-Pygmies**				
N sites	26	26	26	26
Mean (SD)	39.5 (66.9)	307.0 (450.6)	162.0 (123.6)	1283.9 (1004.2)
Min	0.0	0.3	3.1	24.6
Max	298.0	1859.6	456.3	4944.9
*p*^c^	0.10	0.32	0.01	0.23

^a^ P = person

^b^ H = hunter

^c^ *p* values are from one-tailed t tests comparing values for Pygmies and non-Pygmies.

Extraction rates of above 1 animal H^-1^ Yr^-1^ were recorded for 21 and 18 species for non-Pygmy and Pygmy hunters respectively (S2 Table). However, for Pygmies, the highest average extraction rates were typical for only two species, the Peter’s duiker (38.0 ± 58.0 animals H^-1^ Yr^-1^) and the blue duiker (32.3 ± 37.0 animals H^-1^ Yr^-1^). For non-Pygmy hunters, extraction rates were highest for the blue duiker (54.0 ± 51.3 animals H^-1^ Yr^-1^), followed by the bay duiker (17.3 ± 16.5 animals H^-1^ Yr^-1^). Differences in extraction rates for the blue duiker for the two hunter groups were significant (*p* = 0.04; n = 56).

### Extraction per Unit Area

Numbers of animals and biomass hunted per square kilometre per year by Pygmy hunters were significantly lower than for non-Pygmy hunters. On average, a Pygmy hunter took 29.4 ± 31.0 animals km^-2^ Yr^-1^ (a biomass of 487.4 ± 670.5 kg km^-2^ H^-1^ Yr^-1^) whereas a non-Pygmy hunter killed on average 225.7 ± 187.5 animals km^-2^ Yr^-1^ (1730.1 ± 1494.4 kg km^-2^ H^-1^ Yr^-1^). The differences between the two hunter groups were significant (numbers *p* = 0.00; biomass *p* = 0.00).

### Impact on Species

A total of 17 species of those hunted by non-Pygmies were listed in the IUCN Red List as threatened (CR = 1, E = 4; V = 12) whereas only 9 taxa of those hunted by Pygmies were threatened (CR = 1, E = 3, V = 5).

**Fig 5:**
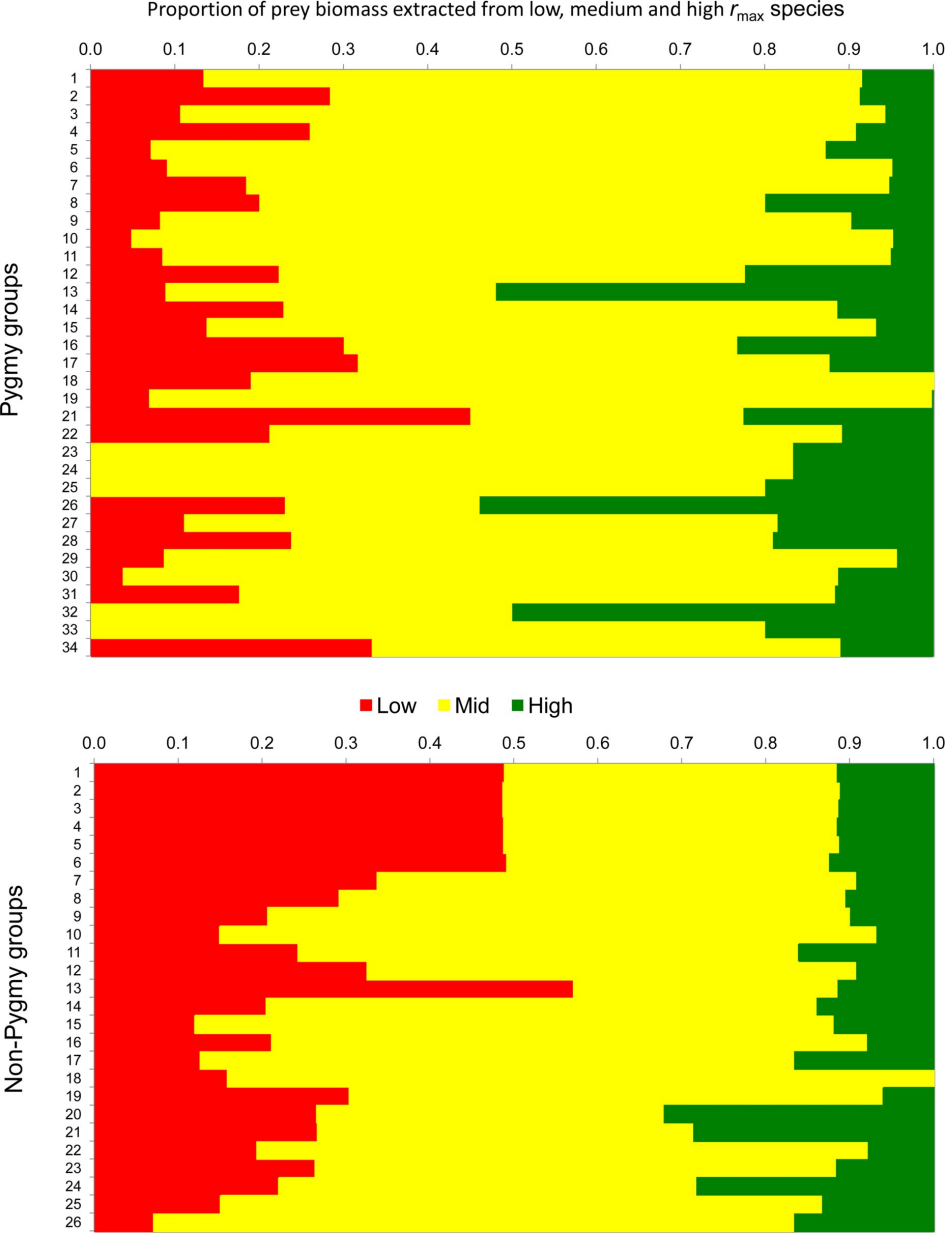
Stacked bar graphs, with sites sorted according to the numbering in Fig 1, of the percentage contribution made by mammals of low, medium and high species population growth rate (*r*_max_) that were hunted by Pygmies and non-Pygmies.

Pygmy settlements derived a much larger proportion of their game biomass from species of medium (0.7 ± 0.2) and high (0.2 ± 0.1) population growth rates, than species with low population growth rates (0.2 ± 0.1) (Fig 5). By contrast, non-Pygmy settlements derived a higher proportion of the biomass harvested from species of low (0.3 ± 0.1) and medium (0.6 ± 0.2) than high (0.1 ± 0.1) population growth rates. Differences between hunter groups in the proportion of species harvested of low and medium population growth rates were statistically significant (low: *p* = 0.00; medium: *p* = 0.00) but not in the proportion of species of high population growth rates. On average, species with medium and high population growth rates accounted for 84% of the biomass harvested in Pygmy and significantly less (73%) in non-Pygmy settlements.

**Fig 6:**
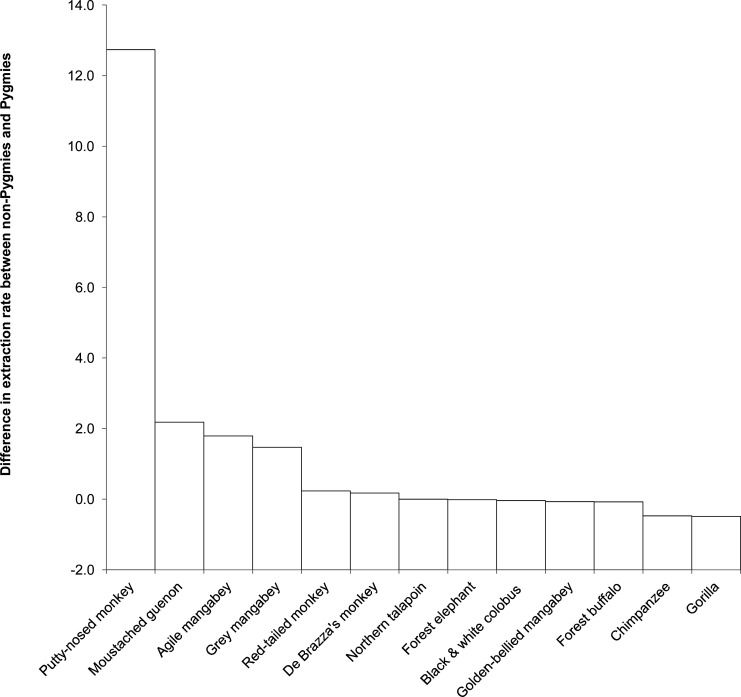
Differences in extraction rates (animal H^-1^ Yr^-1^) for low population growth species hunted by both Pygmies and non-Pygmies.

For the 13 low population growth species common to both Pygmy and non-Pygmy settlements (Fig 6), 7 species were extracted more by Pygmies (difference 0.2 ± 0.2 animals H^-1^ Yr^-1^), the remaining 5 species were extracted more by non-Pygmies (3.1 ± 4.8 animals H^-1^ Yr^-1^).

### Spatial Distribution of Potential Bushmeat Consumption

**Fig 7:**
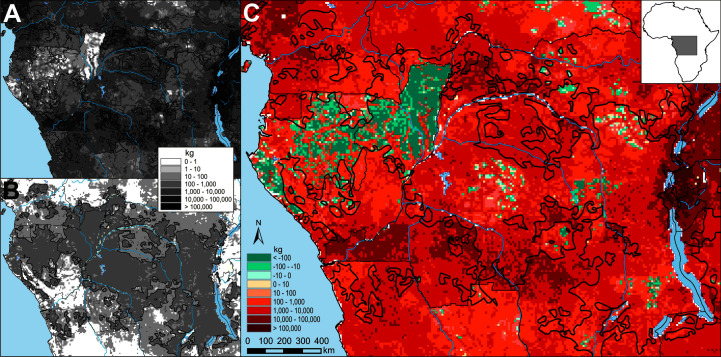
Maps of harvest rates (kg P^-1^ Yr^-1^) for Pygmies and non-Pygmies within the Congo Basin. A. Non-Pygmies; B. Pygmies; C. Differences between Pygmies and non-Pygmies.

The average amounts of bushmeat potentially consumed in each 0.1° × 0.1° grid cell (around 123 km^2^) in the study area were 3,758 kg (maximum 331,136 kg) for non-Pygmies and 77 kg (maximum 159 kg) for Pygmies (Fig 7A and 7B). Differences in harvests between Pygmies and non-Pygmies are shown in Fig 7C. Potential bushmeat consumption by non-Pygmies was significantly higher than for Pygmies throughout the study area, especially in southern Cameroon, northern, southern and eastern DRC, Uganda, Rwanda and Burundi. By contrast, consumption by Pygmy groups was higher than by non-Pygmies in Gabon, northern half of Congo, and scattered areas in the center and east of DRC, where non-Pygmy human populations are extremely low. Around half (46.25%) of all pixels in which Pygmies are found were in non-Pygmy areas of between 1000 and 100,000 kg km^-2^. Total annual potential bushmeat consumption was calculated as 219,044 ± 144,000 tonnes for Pygmies, and 11,619,172 ± 6,888,000 tonnes for non-Pygmy populations.

## Discussion

Harvest profiles obtained here used different sampling methods, including daily or weekly interviews and monitoring of hunting forays and kills brought back from the forest at the settlement or household level. Despite the variety of methods employed in the assembled studies, often involving observers of different backgrounds, including local field assistants, anthropologists, and wildlife biologists, our sample, arguably the most extensive to date, can be used to determine differences in hunting of wild animals by Pygmies and non-Pygmies. Overall, Pygmies hunted a smaller range of taxa but took a higher proportion of prey of a greater mean body mass than non-Pygmies. Harvest rates, animals per inhabitant, were almost twice as high in non-Pygmy sites than in Pygmy sites, as were extraction rates. There were no significant differences in biomass values, due to the higher body mass of species hunted by Pygmies. However, when converted to extraction per hunter km^-2^, non-Pygmy sites harvested more per unit area than Pygmy groups. Moreover, mapped bushmeat consumption across the Congo Basin indicated that non-Pygmies are likely to be responsible for 27 times more bushmeat consumed than Pygmies.

Our results clearly indicate that the two hunter groups use the wildlife resource in different ways and their hunting impact is substantially different. A partial explanation for these contrasts is related to how hunting is carried out by each hunter group. Thus, in circumstances where Pygmies are hunting for subsistence, and if not contracted by outsiders to hunt for commercial purposes [36], they employ more traditional projectile-type methods (bow and arrow, crossbow) or nets and spears, than snares or firearms. Thus, typically, animals hunted by Pygmies i.e. more red duikers, less primates, large rodents and carnivores, can be accounted for by the vulnerability of these species groups to the type of hunting techniques used. Prey profiles resulting from the typical net hunting practiced by the Mbuti [37–38], differ from that of the sympatric Efe, who do not practice net hunting, preferring bows and arrows [36,39, 40]. However, the use of cable snares alongside nets for hunting as employed by the Aka [41–46] result in a different set of prey being hunted compared to collective spear hunting by the Baka [47,48]. In addition to vulnerability of species to these different hunting techniques practiced by Pygmies compared to non-Pygmies, it is probable that Pygmies’ ecological knowledge and hunting skills allows them to successfully target larger species. Thus, in more intact habitats, Pygmies may target a narrower range of preferred species regardless of their local abundance since larger prey yields the greatest amount of meat per unit of energy or time allocated [49–54]. Despite this, the number of very large prey of >100 kg (forest buffalo, bongo, giant forest hog, okapi, and forest elephant) hunted by Pygmies was significantly lower than for non-Pygmy hunters. This observation may be explained by the use of traditional weapons by Pygmy hunters, not just because these may be preferred, but also due to their more limited access to shotguns and rifles [36, 49]. However, in situations where Pygmies are able to use guns, reported hunting success rates can be higher than for non-Pygmies. For example, in a study in northern Congo, Pygmies captured four times more medium-large ungulates as non-Pygmies using the same hunting technique [55].

In this paper, we argue that as anthropogenic pressures increase (defined by increasing human populations), changes in faunal assemblages are reflected in the mean body mass of animals hunted. We show here that, as expected, there is an overall negative relationship between the mean body mass of mammals hunted and human population numbers. However, there are differences between Pygmy and non-Pygmy sites. According to the data available, we show that Pygmy sites can be found in a wider variety of ecological circumstances than non-Pygmy sites, ranging from deep forest to modified habitats where human population densities are high. Non-Pygmy sites are only found in modified habitats within medium to high human population densities.

Because some Pygmy groups are still found in areas of relatively low non-Pygmy populations and presumably more intact faunas, the mean body mass of hunted mammals is appreciably larger compared to those for areas where humans are more numerous. This relationship between human population and prey depletion profiles has been demonstrated in previous studies [32]. Nonetheless, in areas where fauna is much depleted (inferred by the higher human numbers), recorded mean body masses in Pygmy settlements were much lower than those estimated for non-Pygmy sites. Although it is difficult to make direct comparisons, it is possible that Pygmy hunters in high human population densities may be disadvantaged because larger prey are severely depleted [36]. As observed in this study, the significant difference between Pygmies and non-Pygmies in the proportion of blue duikers hunted relative to red duikers may be because most Pygmy sites are situated in less disturbed areas compared to non-Pygmy sites. Blue duikers are more common in areas under continuous heavy hunting pressure, as suggested by Yasuoka [47], because blue duikers respond better to higher hunting pressure than red duikers, due to their overall higher intrinsic rate of population increase.

The general picture that emerges from our study is that Pygmy hunters have less impact on game animals in the Congo Basin than the more numerous, more generalist, non-Pygmy hunters. Basin-wide studies that have compared production and extraction levels for mammal species [18] have already indicated that bushmeat hunting may be responsible for the removal of more than twice the likely production of these species in the Congo Basin. Fa et al. [18] and later Nasi et al. [19] calculated annual extraction levels of around 5 million t Yr^–1^ of mammal meat in the Congo Basin, a figure that contrasts with the more than 11 million t Yr^–1^ for all terrestrial vertebrates estimated in the present study. At such a large geographical scale, and with the probable limitations of the available data, it is difficult to assess the accuracy of these calculations. However, despite this, our study uses similar analyses to measure hunting profiles of Pygmies and non-Pygmies, and therefore is important because it differentiates the impact of overlapping hunter groups in Central Africa. From our data, we are not able to infer whether hunting by either of the two hunter groups is sustainable. Nonetheless, studies at a more local scale, at least for the more commonly hunted duikers, suggest that hunting by Pygmies [43, 45, 56] and non-Pygmies [30] is often not sustainable. This is not unexpected since subsistence game hunting can often have profound negative effects on the species diversity, standing biomass, and size structure of vertebrate assemblages in tropical forests. This occurs mainly through local population declines, if not extirpation, of large-bodied vertebrate taxa, which make a disproportionately large contribution to non-hunted forests in terms of their aggregate biomass and role in ecosystem functioning. As a consequence of the heavier hunting pressure especially on large-bodied mammals, gradual, if not sudden, population declines can be expected for these species because they bear the brunt of the initial offtake and tend to recover slowly from a selective harvest, mainly due to low reproductive rates [55]. These population declines will reach a threshold whereby exploiting a greater range of smaller, less-preferred species will result in a higher return, thus shifting prey selection to these species. In addition to the potential ecological impact of a shift in hunt profile, social-cultural institutions governing the division of bushmeat can vary depending on the overall hunt harvest and prey items, meaning that divisions of meat between hunters, buyers and sellers and within households [45]. This combined with the loss of certain mammals with high energy content in their meat (e.g. red-river hog *Potamochoerus porcus* and giant forest hog *Hylochoerus meinertzhageni*) is likely to have a knock-on effect on those hunting groups more reliant on this food source.

Although subsistence hunters can have a negative impact on prey populations, it is the shift to commercial hunting that will have the greatest impact on the Congo Basin vertebrate fauna. Commercialization of hunting is not limited to non-Pygmy groups [56, 57], although as we have seen in our study the proportion of game sold is significantly higher than that seen in Pygmy settlements. However, the increase in consumption and value of bushmeat in many regions in Central Africa has occurred because of increased demand from urban areas. Subsistence hunting and fishing have usually not posed a significant threat to the abundant wildlife species living around rural forest communities. But as urban centres grow, the commercial bushmeat trade to supply them poses an ever-increasing threat, both to the animals of the forest and the people who have hunted them. The urbanization of most of Africa is moving quickly forward, especially south of the Sahara. While only one in 10 people lived in urban areas in 1900, almost half of all sub-Saharan inhabitants now live in towns and cities [58]. This demographic change has dramatically altered the way people produce and obtain their food. It places increasing pressure on food production systems, which can have far-reaching impacts, both on domesticated and wild environments. The growing number of bushmeat markets in Central Africa is a direct result of the population shift to urban centres. In urban markets, bushmeat also complements the under-supply of domestic meat sources—cattle cannot be raised in the central African forest region because of trypanosomiasis. Many recent migrants to cities who were accustomed to eating bushmeat may prefer the taste of bushmeat, consuming bushmeat as a luxury, rather than a dietary need. Others may purchase the cheapest meat available, considering bushmeat to be a normal good [59–60]. The burgeoning urban population fuels an ever-increasing, lucrative trade of wild animals from rural and protected areas. This trade is now the most significant immediate threat to the future of wildlife in Africa and around the world.

In other parts of the world, conservationists have used moral and ethical, as well as ecological arguments [61–63] to ally themselves with indigenous peoples and to promote indigenous land titling, co-management of protected areas, and use rights in protected area buffer zones [64–66]. Central African countries, however, do not recognize indigenous peoples nor grant them exclusive communal land and resource rights. Settlement and hunting are prohibited inside protected areas, while only customary use rights are recognized for both Pygmy and non-Pygmy peoples outside protected areas. Buffer zones or multi-use areas outside some strictly protected areas allow subsistence hunting by local residents [67–80].

Natural resources management can only link biodiversity conservation to the needs of local people if crucial resources are not overexploited to the point of collapse. The perceived value of the forest to indigenous people can therefore become considerably reduced as game populations are overexploited or even driven to local extinction. This study shows they generally exert less pressure on wildlife than do non-indigenous populations, therefore are more likely allies for conservationists—as indigenous groups in Latin America are. This will also strengthen the joint cause of conservationists and indigenous-rights advocacy groups for maintaining large tracts of forests against encroachment by more insidious interests, particularly commercial hunting, but also extractive industries and commercial agri-business. Further studies on the impacts of commercial hunting within and around protected areas, and of the transportation and export routes for bushmeat to urban centres are required to implement appropriate wildlife management programs that ensure the preservation of both biological and cultural diversity in the Congo Basin. As policy mechanisms to allow hunters and rural communities rights over forest and wildlife management are relatively undeveloped compared to other parts of the continent [80], opportunities for developing appropriate policy mechanisms or implementing existing mechanisms could be sought. It is possible that areas where a CBNRM type approach might have more traction will be in areas with relatively high proportion of Pygmy hunters in relation to non-Pygmies, and relatively low levels of commercial bushmeat extraction. Importantly, the participation of Pygmies in facilitating and participating in the hunting for a commercial pool [36] within CBNRM areas needs to be controlled.

## Supporting Information

S1 Table Summary information for the studies on the 60 settlements compiled here, numbered as in Fig 1. (PDF)

S2 Table File containing data on the list of species hunted, by sample sites and ethnic group, used in this paper. (XLS)

## References

[1] Lewis J. BaYaka Pygmy multi-modal and mimetic communication traditions. In: Dor D, Knight C, Lewis J, editors. The social origins and evolution of language. Studies in the evolution of language. Oxford: Oxford University Press; 2014. pp. 77–91.

[2] Patin E, Laval G, Barreiro LB, Salas A, Semino O, Santachiara-Benerecetti S, et al. Inferring the demographic history of African farmers and pygmy hunter–gatherers using a multilocus resequencing data set. PLoS Genet 2009; 5(4): e1000448. 10.1371/journal.pgen.1000448 19360089 PMC2661362

[3] Vansina J. New linguistic evidence on the expansion of Bantu. J Afr Hist 1995; 36:173–195.

[4] Brechin SR., Wilshusen PR, Fortwangler CL, West PC. Contested nature. Promoting international biodiversity with social justice in the twenty-first century. State University of New York Press, Albany; 2003.

[5] Adams WM, Hutton J. People, parks and poverty: political ecology and biodiversity conservation. Conserv Soc 2007; 5: 147–183.

[6] Berkes F. Community-based conservation in a globalized world. Proc Natl Acad Sci U S A 2007; 104: 15188–15193. 17881580 10.1073/pnas.0702098104 PMC2000555

[7] McShane TO, Hirsch PD, Trung TC, Songorwa AN, Kinzig A, Monteferri B, et al. Hard choices: making tradeoffs between biodiversity conservation and human well-being. Biol Conserv 2011; 144: 966–972.

[8] Brockington D. Fortress conservation: the preservation of the Mkomazi Game Reserve, Tanzania. Indiana University Press, Bloomington, Indiana; 2002.

[9] Wilshusen PR. Exploring the political contours of conservation: A conceptual view of power in practice. In: Brechin SR, Wilshusen PR, Fortwangler CL, West PC, editors. Contested nature: promoting international biodiversity with social justice in the twenty-first century 2003; SUNY Press, Albany, NY. pp. 41–58.

[10] Sarkar S, Montoya M. Beyond parks and reserves: The ethics and politics of conservation with a case study from Perú. Biol Conserv 2011; 144: 979–988.

[11] IUCN. The world conservation strategy. Geneva, International Union for Conservation of Nature and Natural Resources, United Nations Environment Programme, World Wildlife Fund; 1980.

[12] United Nations. The Universal Declaration of Human Rights (United Nations, New York); 1948. Available: http://www.un.org.en/documents/udhr/.

[13] Pyhälä A., Osuna Orozco A, Counsell S. Protected areas in the Congo basin: failing both people and biodiversity? Rainforest Foundation-UK, London; 2016.

[14] Curran B, Sunderland T, Maisels F, Oates J, Asaha S, Balinga M, et al. Are Central Africa's protected areas displacing hundreds of thousands of rural poor? Conserv Soc 2009; 7: 30–45.

[15] Bennett EL, Robinson JG. Hunting of wildlife in tropical forests: implications for biodiversity and forest peoples. International Bank for Reconstruction/The World Bank, Washington DC; 2000.

[16] Fa JE, Ryan SF, Bell DJ. Hunting vulnerability, ecological characteristics and harvest rates of bushmeat species in Afrotropical forests. Biol Conserv 2005; 121: 167–176.

[17] Wilkie DS, Carpenter JF. Bushmeat hunting in the Congo Basin: An assessment of impacts and options for mitigation. Biodiv Conserv 1999; 8: 927–955.

[18] Fa JE, Peres CA, Meeuwig J. Bushmeat exploitation in tropical forests: an intercontinental comparison. Conserv Biol 2002; 16: 232–241. 10.1046/j.1523-1739.2002.00275.x 35701970

[19] Nasi R, Taber A, van Vliet N. Empty forests, empty stomachs: bushmeat and livelihoods in Congo and Amazon Basins. Int Forestry Rev 2011; 3: 355–368.

[20] Robinson JG, Bennett E. Hunting for sustainability in tropical forests. Columbia University Press, New York; 2000.

[21] Bennett EL. Is there a link between wild meat and food security? Conserv Biol 2002; 16: 590–92.

[22] Olivero J, Fa JE, Farfán MA, Lewis J, Hewlett B, Breuer T, et al. Distribution and numbers of Pygmies in Central African forests. PLoS ONE 2016; 11(1): e0144499. 10.1371/journal.pone.0144499 26735953 PMC4711706

[23] White F. The vegetation of Africa–A descriptive memoir to accompany the UNESCO/AETFAT/UNSO vegetation map of Africa. Series “Natural Resources Research” XX, Paris, UNESCO; 1983.

[24] IUCN. The IUCN red list of threatened species. Version 2015–4. Available: http://www/iucnredlist.org.

[25] Kingdon J, Happold D, Butynski T, Hoffmann M, Happold M, Kalina J. Mammals of Africa Volumes I-VI. London: Bloomsbury Natural History; 2013.

[26] Dunning JB Jr. CRC Handbook of avian body masses, Second Edition. CRC Press, Boca Raton, Florida; 2007.

[27] Ingram DJ, Coad L, Collen B, Kümpel NF, Breuer T, Fa JE, et al. Indicators for wild animal offtake: methods and case study for African mammals and birds. Ecol. Soc. 20(3):40. 10.5751/ES-07823-200340; 2015.

[28] Redford KH, Robinson JG. The game of choice: patterns of Indian and colonist hunting in the Neotropics. Amer Anthro 1987; 89: 650–667.

[29] Hoare AL. Resource rights and timber concessions: Integrating local peoples’ land-use practices in forest management in the Congo Basin. Rainforest Foundation-UK, London; 2007.

[30] Bobo KS, Kamgaing TOW, Kamdoum EC, Dzefack ZCB. Bushmeat in southeastern Cameroon: magnitude and impact on duikers (*Cephalophus* spp.). Afr Study Monogr Supplementary Issue 2015; 51: 119–141.

[31] Fa JE, Olivero J, Farfán MÁ, Márquez AL, Vargas JM, Real R, et al. Integrating sustainable hunting in biodiversity protection in Central Africa: hot spots, weak spots, and strong spots. PLoS ONE 2014; 9(11): e112367. 10.1371/journal.pone.0112367 25372705 PMC4221474

[32] Fa JE, Olivero J, Farfán MÁ, Márquez AL, Duarte J, Nackoney J, et al. Correlates of bushmeat in markets and depletion of wildlife. Conserv Biol 29: 805–815; 2015. 10.1111/cobi.12441 25580729

[33] Schneider A, Friedl MA, Potere D. A new map of global urban extent from MODIS data. Environ Res Let 2009; 4: 044003.

[34] Schneider A, Friedl MA, Potere D. Monitoring urban areas globally using MODIS 500m data: new methods and datasets based on urban ecoregions. Remote Sens Environ 2010; 114: 1733–1746.

[35] Friedl MA, Sulla-Menashe D, Tan B, Schneider A, Ramankutty N, Sibley A. MODIS Collection 5 Global Land Cover: algorithm refinements and characterization of new datasets. Remote Sens Environ 2010; 114: 168–182.

[36] Riddell M. Assessing the impacts of conservation and commercial forestry on livelihoods in northern Republic of Congo. Conserv Soc 2013; 11: 199–217.

[37] Harako R. The Mbuti as hunters: a study of ecological anthropology of the Mbuti Pygmies. Kyoto University African Studies 1976; 10: 37–99.

[38] Tanno T. The Mbuti net-hunters in the Ituri forest, eastern Zaire: their hunting activities and band composition. Kyoto University African Studies 1976; 10: 101–135.

[39] Ichikawa M. An examination of the hunting-dependent life of the Mbuti pygmies, eastern Zaire. Afr Study Monogr 1983; 4: 55–76.

[40] Carpaneto GM, Germi F. The mammals in the zoological culture of the Mbuti Pygmies in north-eastern Zaire. Hystrix 1989; 1: 1–83.

[41] Terashima H. Mota and other hunting activities of the Mbuti archers: A socio-ecological study of subsistence technology. Afr Study Monogr 1983; 3: 71–85.

[42] Bahuchet S. Les Pygmées Aka et la forêt centrafricaine. Ethnologie écologique. Paris: SELAF; 1985.

[43] Takeuchi K. Subsistence hunting in African tropical forest: Hunting technics and activities among the Aka hunter-gatherers, northeastern Congo. (in Japanese). Zoo-archaeology 1995; 4: 27–52.

[44] Kitanishi K. Seasonal changes in the subsistence activities and food intake of the Aka hunter-gatherers in northeastern Congo. Afr Study Monogr 1995; 16: 73–118.

[45] Noss AJ. Cable snares and nets in the Central African Republic. In: Robinson JG, Bennett EL, editors. Hunting for sustainability in tropical forests. Columbia University Press. New York; 2000. pp. 282–304.

[46] Lupo K, Schmitt DN. Small prey hunting technology and zooarchaeological measures of taxonomic diversity and abundance: Ethnoarchaeological evidence from Central African forest foragers. J Anthro Archaeo 2005; 24: 335–353.

[47] Yasuoka H. The sustainability of duiker (*Cephalophus* spp.) hunting for the Baka hunter-gatherers in southeastern Cameroon. African Study Monographs Supplementary Issue 2006; 33: 95–120.

[48] Bahuchet S. History of the inhabitants of the Central African rain forest: perspectives from comparative linguistics. In: Hladik CM, Hladik A, Linares OF, Pagezy H, Semple A, Hadley M, editors. Man and Biosphere Series, Vol 13. UNESCO, Parthenon, Paris/Lancs. 1993; pp. 37–54.

[49] Riddell M. Hunting and rural livelihoods in northern Republic of Congo: local outcomes of integrated conservation and development. PhD Thesis, University of Oxford; 2011.

[50] Hawkes K, Hill K, O’Connell J. Why hunters gather: Optimal foraging and the Aché of eastern Paraguay. Am Ethnologist 1982; 2: 379–398.

[51] Vickers W. An analysis of Amazonian hunting yields as a function of settlement age. Working Papers on South American Indians 1980; 2: 7–29.

[52] Bodmer RE. Managing Amazonian wildlife: biological correlates of game choice by detribalized hunters. Ecol Appl 1995; 5: 872–877.

[53] Fa JE, Peres CA. Game vertebrate extraction in African and Neotropical forests, an intercontinental comparison. In Reynolds JD, Mace GM, Robinson JG, Redford KH editors. Conservation of Exploited Species, Cambridge; 2001. pp. 203–224.

[54] Yasuoka H. Snare hunting among Baka hunter-gatherers: Implications for sustainable wildlife management Afr Study Monogr, Suppl. 2014; 49: 115–136.

[55] Jerozolimski A, Peres CA. Bringing home the biggest bacon: a cross-site analysis of the structure of hunter-kill profiles in Neotropical forests. Biol Conserv 2003; 111: 415–425.

[56] Hart JA. Impact and sustainability of indigenous hunting in the Ituri forest, Congo-Zaire: a comparison of hunted and unhunted duiker populations. In Robinson JG, Bennett EL, editors. Hunting for sustainability in tropical forests. New York: Columbia University Press; 2000. pp. 106–155.

[57] Hart JA. From subsistence to market: A case study of the Mbuti net hunters. Hum Ecol 1978; 6: 325–353.

[58] United Nations, Department of Economic and Social Affairs, Population Division. World urbanization prospects: The 2014 revision, highlights (ST/ESA/SER.A/352); 2014.

[59] Kümpel NF, East T, Keylock N, Rowcliffe JM, Cowlishaw G, Milner-Gulland EJ. Determinants of bushmeat consumption and trade in Rio Muni, Equatorial Guinea: an urban-rural comparison. In: Davies G, Brown D, editors. Bushmeat and livelihoods: wildlife management and poverty reduction. Oxford: Blackwell Publishing; 2007. pp.73–91.

[60] Van Vliet N, Nebedde C, Gambalemoke S, Akaibe D, Nasi R. The bushmeat market in Kisangani, Democratic Republic of Congo: implications for conservation and food security. Oryx 2012; 46: 196–203.

[61] Beltrán J. Indigenous and traditional peoples and protected areas: principles, guidelines and case studies. Gland, Switzerland and Cambridge, UK: IUCN and WWF International; 2000.

[62] Conservation International. Indigenous peoples and Conservation International: Principles for partnerships. Available: https://www.conservationgateway.org/Documents/21_CI%20IP%20Principles.pdf.

[63] United Nations. Declaration on the rights of indigenous peoples. Resolution adopted by the General Assembly. 61/295; 2007.

[64] WWF. Indigenous peoples and conservation: WWF statement of principles. Gland: WWF-International; 1996.

[65] Arambiza E., Painter M. Biodiversity conservation and the quality of life of indigenous people in the Bolivian Chaco. Hum Organ 2006; 65: 20–34.

[66] Redford KH, Mansour JA. (editors) Traditional peoples and biodiversity conservation in large tropical landscapes. America Verde Publications, The Nature Conservancy; 1996.

[67] Redford KH, Painter M. Natural alliances between conservationists and indigenous peoples. Working Paper No. 25. New York, WCS; 2006.

[68] Redford KH, Stearman AM. Forest-dwelling native Amazonians and the conservation of biodiversity: interests in common or in collision? Conserv Biol 1993; 7: 248–255.

[69] Bikaba D. Indigenous people and the Kahuzi-Biega National Park in the Democratic Republic of the Congo. In: Painemilla KW, Rylands AB, Woofter A, Hughes C, editors. Indigenous peoples and conservation: from rights to resource management. Washington, DC: Conservation International. 2010; pp. 49–59.

[70] Bobo KS, Weladji RB. Wildlife and land use conflicts in the Mbam and Djerem Conservation Region, Cameroon: status and mitigation measures. Hum Dimens Wildl 2011; 16: 445–457.

[71] Congo Biodiversity Initiative. Available: http://www.congobiodiv.org/en.

[72] Mavah GA. Integrating rural people into natural resources and wildlife management in the Republic of the Congo. Ph.D. Dissertation Gainesville, FL, USA: University of Florida; 2015.

[73] Mbomio Ngomo D, Ngua Ayecaba G. Plan de Manejo Río Campo. Bata: ANDEGE; 2009.

[74] Nelson J, Hossack L. (editors). Indigenous peoples and protected areas in Africa: from principles to practice. Moreton-in-Marsh: Forest Peoples Programme, UK; 2003.

[75] Redford KH, Fearn E. (editors). Protected areas and human displacement: a conservation perspective. Working Paper #29. New York, Wildlife Conservation Society; 2007.

[76] Schmidt-Soltau K. Plan de développement des peuples autochtones. Programme Sectoriel Forets et Environnement, Ministère de l’Economie Forestière, des Eaux, de la Pèche, de l’Environnement chargé de la Protection de la Nature: République Gabonaise. Libreville, Gabon; 2005.

[77] Usongo L, Tchikangwa Nkanje B. Participatory approaches towards forest conservation: the case of Lobéké National Park, south east Cameroon. Int J Sust Dev World 2004; 11: 119–127.

[78] Virunga National Park. Available: http://www.virunga.org.

[79] Wildlife Conservation Society-Congo Brazzaville. Lac Télé community reserve. Available: http://www.wcscongo.org/Wild-Places/Lac-T%C3%A9l%C3%A9-Community-Reserve.aspx.

[80] Roe D, Nelson F, Sandbrook C. Community management of natural resources in Africa: Impacts, experiences and future directions. London, UK: International Institute for Environment and Development; 2009.

