# Supplementary material for: Differences between Pygmy and Non-Pygmy Hunting in Congo Basin Forests
Source: PLoS One. 2016 Sep 2;11(9):e0161703. doi: 10.1371/journal.pone.0161703 (PMC5010184; doi:10.1371/journal.pone.0161703)
Supplement: S1 Table — (PDF) [file pone.0161703.s001.pdf]

S1 Table. Summary information for the studies on the 60 settlements compiled here, numbered as in Fig.1

Site No.	Country	Region	Village/camp	Study year/s	Study days	Population	No. of hunters	Hunting techniques[1]	% sold	Data Source
**Pygmies**										
1	CM	Boumba-Ngoko, East Province	Malea Ancien	2002	212	118	25	CS, H, M, S	25	Hattori, this study
2	CM	Boumba-et-Ngoko, East Province	Camp, 40 km SSE Zoulabot Ancien	2002	90	89	12	CS, H, M, S, SG	0	Yasuoka (2006a)
3	CM	Boumba-Ngoko, East Province	Camp, 21.5 km SSE Zoulabot Ancien	2002/2003	71	23	5	CS	75	Yasuoka (2006b)
4	CM	Boumba-Ngoko, East Province	Camp, 19 km SSE Zoulabot Ancien	2003	83	11	2	CS	75	Yasuoka (2006b)
5	CM	Boumba-Ngoko, East Province	Camp, 18 km SSE Zoulabot Ancien	2003	91	9	2	CS	75	Yasuoka (2006b)
6	CM	Boumba-Ngoko, East Province	Camp, 9.5 km S Zoulabot Ancien	2003	85	8	3	CS	75	Yasuoka (2006b)
7	CM	Boumba-Ngoko, East Province	Camp, 8 km SE Zoulabot Ancien	2003	79	12	3	CS	75	Yasuoka (2006b)
8	CM	Boumba-Ngoko, East Province	Camp, 40 km SSE Zoulabot Ancien	2005	32	110	17	CS, H, M, S	0	Yasuoka (2009)
9	CM	Boumba-Ngoko, East Province	Camp, 12 km S Zoulabot Ancien	2012	49	35	7	CS	0	Yasuoka et al. (2015)
10	CM	Boumba-Ngoko, East Province	Camp, 21 km S Gribé	2014	33	15	6	CS	90	Yasuoka et al. (2015)
11	CM	Boumba-Ngoko, East Province	Camp, 17 km S Gribé	2014	19	29	4	CS	40	Yasuoka et al. (2015)
12	CM	Haut-Nyong, East Province	Village, 45km E Lomie	2013	60	156	80	CS, SG, S, M, O	-	Duda & Gallois, this study
13	CM	Haut-Nyong, East Province	Village, 12km S Messok	2013	60	110	87	CS, SG, S, M, O	-	Duda & Gallois, this study
14	CM	Boumba-Ngoko, East Province	Camp S, 50 km W Moloundou	2001/2002	35	24	5.8	CS, G, H, M, S	0	Hayashi (2008)
15	CM	Boumba-Ngoko, East Province	Camp S2, 50 km W Moloundou	2001/2003	59	32	5.4	CS, G, H, M, S	0	Hayashi (2008)
16	CM	Boumba-Ngoko, East Province	Migratory camp, 50 W Moloundou	2001/2004	28	32	7.6	CS, G, H, M, S	0	Hayashi (2008)
17	CG	Sangha region	Mielekouka	2009/2010	180	400	20	CS, SG	-	Mbete et al. (2010)
18	CG	Likouala region	Makao-Linganga	2007	80	670	104	S, SG	77	Riddell (2013)
19	CG	Odzala National Park	Eyoka neighbourhood	1992/1993	160	117	58	B, CS, H, O, SG, T	0	Carpanetto (1994)
20	CF	Dzanga-Sangha Special Reserve	Mossapoula	1994/1995	90	320	195	CS, N	-	Noss (2000)
21	CF	N'gotto Forest Reserve	Camp, near Ndele and Grima villages	1999/2000, 2001/2002	218		69	-	-	Lupo & Schmitt (2005)
22	CD	Ituri Foret	Camp, N Nduje	1982/1983	368	283	-	B, CS, H, N, SG	-	Wilkie (1987)

23	CD	Ituri Forest, near Bayenga	Alipanda Camp	1985	5	22	8	N, T	0	Carpanetto & Germi, this study
24	CD	Ituri Forest, near Nduye	Bataka camp	1985	4	17	6	B, T	0	Carpanetto & Germi, this study
25	CD	Ituri Forest, Bafwasende/Bomili	Bafwabwane camp	1985	7	28	11	B, N, O, S, T	35	Carpanetto & Germi, this study
26	CD	Ituri Forest, near Nduye	Apa Njaro Camp	1985	6	34	17	B, O, S, T	10	Carpanetto & Germi (1989)
27	CD	Ituri Forest, Epulu/Nduye	Apa Kengetu Camp	1985	8	27	14	N, O, S, T	40	Carpanetto & Germi (1989)
28	CD	Ituri Forest, near Bafwasende	Bafwaguda Camp	1985	4	21	11	N, O, S, SG, T, V	70	Carpanetto & Germi (1989)
29	CD	Ituri Forest, near Bomili	Angbetima Camp	1985	3	29	22	B, N, O, S, T	40	Carpanetto & Germi (1989)
30	CD	Ituri Forest, near Epulu	Apa Mutelepu Camp	1987	10	38	18	N, O, S, T	40	Carpanetto & Germi (1989)
31	CD	Ituri Forest, near Epulu	Makongo Tudu Camp	1985	10	27	14	B, CS, N, O, S, T	40	Carpanetto & Germi, this study
32	CD	Ituri Forest, near Nduye	Apa Andelifou Camp	1988	3	30	12	B, S, T, O	0	Carpanetto & Germi, this study
33	CD	SW of Matango, near Beni	Apa Kungubu Camp	1988	3	18	3	B, H, N, S, T	0	Carpanetto & Germi, this study
34	CD	Ituri Forest, near Epulu	Apa Tito Camp	1988	5	17	5	B, N, O, S, T	30	Carpanetto & Germi, this study
**Non-Pygmies**										
35	CM	Korup National Park	Erat	1988	365	175	63	CS, SG	75	Infield (1988)
36	CM	Korup National Park	Ekundu-Kundu	1988	365	90	22.5	CS, SG	95	Infield (1988)
37	CM	Korup National Park	Bera	1988	365	60	11.4	CS, SG	89	Infield (1988)
38	CM	Korup National Park	Esukutan	1988	365	115	55.2	CS, SG	82	Infield (1988)
39	CM	Korup National Park	Ikenge	1988	365	100	36	CS, SG	94	Infield (1988)
40	CM	Korup National Park	Bareka-Batanga	1988	365	35	11.2	CS, SG	-	Infield (1988)
41	CM	Dja Biosphere Reserve	Mekas	1994/1995	300	368	14	CS, SG	65.5	Muchaal and Ngandjui (1999)
42	CM	Dja Biosphere Reserve	Kompia	1996	210	96	14	CS, SG	68	DelVingt (1997)
43	CM	Dja Biosphere Reserve	Ekom	1996	240	560	15	CS, SG	40	DelVingt (1997)
44	CM	Dja Biosphere Reserve	Malen	1997	150	500	11	CS, SG	-	Fotso & Ngnegueu (1998)
45	CM	Boumba-Bek National Park	Masséa	2013	7.67	1360	8	CS, SG	82	Bobo et al. (2014)
46	CM	Boumba-Bek National Park	Bintom	2013	9.58	105	12	CS, SG	69.2	Bobo et al. (2014)
47	CM	Boumba-Bek National Park	Zoka Diba	2013	7.75	840	16	CS, SG	68.7	Bobo et al. (2014)
48	CM	Boumba-Bek National Park	Gribé	2013	7.29	575	38	CS, SG	67.6	Bobo et al. (2014)

49	CM	Boumba-Bek National Park	Song Ancien	2013	7.91	11	11	CS, SG	61.9	Bobo et al. (2014)
50	CM	Boumba-Bek National Park	Gouonepoum Ancien	2013	7	7	7	CS, SG	58.1	Bobo et al. (2014)
51	CM	Boumba-Bek National Park	Zoulabot Ancien	2013	9	8	9	CS, SG	56	Bobo et al. (2014)
52	CM	Boumba-Bek National Park	Malea Ancien	2013	12	123	12	CS, SG	63.2	Bobo et al. (2014)
53	CM	Boumba-Bek National Park	Ngatto Ancien	2013	9	242	9	CS, SG	69.4	Bobo et al. (2014)
54	CM	Banyang-Mbo Wildlife Sanctuary	Banyangi (20 villages)	1999/1999	1020	6594	46	CS, SG	32	Willcox & Nambu (2007)
55	CM	Banyang-Mbo Wildlife Sanctuary	Mbo (20 villages)	1999/2000	1020	1959	50	CS, SG	11	Willcox & Nambu (2007)
56	CM	Campo District	Nkoelon	1989/1990	395	60	14	CS, SG	-	Dounias (1999)
57	GA	Ogooue-Ivindo Province	Makokou	1988	365	130	76	CS, SG	59	Lahm (1993)
58	GQ	Rio Muni	Sendje	1998/1999	365	1500	42	CS, SG	67	Fa and Garcia Yuste (2001)
59	GQ	Rio Muni	Sendje	2002/2004	457	1500	40	CS, SG	-	Kumpel (2006)
60	CF	Dzanga-Sangha Special Reserve	Bayanga	1994	480	2500	17	CS, SG	-	Noss (1995, 1998)

Data sources

Carpaneto GM. Parc National d'Odzala: Ethnozoologie, Faune et Ecotourisme. Rapport Final. ECOFAC/AGRECO-CTFT, Brussels, Belgium; 1994.

Carpaneto GM, Germi F. The mammals in the zoological culture of the Mbuti Pygmies in north-eastern Zaire. Hystrix 1989; 1: 1-83.

Bobo, KS, Kamgaing TOW, Ntumwel BC, Kagalang D, Kengne PNJ, Ndengue SML, Badjeck MMN, Aghomo FFM. Species richness, spatial distributions and densities of large- and medium-sized mammals in the northern periphery of Boumba-Bek National Park, southeastern Cameroon. Afr Study Monogr Supplementary Issue 2014; 49: 91-114.

Delvingt W. La chasse villageoise synthèse régionale des études réalisées durant la première phase du programme ECOFAC au Cameroun, au Congo et en République Centrafricaine. ECOFAC/AGRECO-CTFT, Brussels, Belgium; 1997.

Dounias E. Le câble pris au piège de la conservation. Technologie du piégeage et production cynégétique chez les Mvae du sud Cameroun forestier. In: Bahuchet S, Bley D, Pagezy H, Vernazza-licht N. editors. L'homme et la forêt tropicale. Travaux Société Ecologie Humaine, Paris; 1999. pp. 281–300.

Fa JE, Garcia Yuste JE. Commercial bushmeat hunting in 395 the Monte Mitra forests, Equatorial Guinea: extent and impact. Anim Biodivers Conserv 2001; 24: 31–52.

Fotso RC, Ngnegueu PR. 1998. Commercial hunting and its consequences on the dynamics of duiker populations. ECOFAC/Cameroon.

Hayashi K. Hunting activities in forest camps among the Baka hunter-gatherers of southeastern Cameroon. Afr Study Monogr 2008; 29: 73-92.

Infield M. Hunting, trapping and fishing in villages within and on the periphery of the Korup National Park. WWF Publication 436 3206/A9.6, Goldaming; 1988.

Kümpel NF. Incentives for sustainable hunting of bushmeat in Río Muni, Equatorial Guinea. PhD thesis, Institute of Zoology, Zoological Society of London and Imperial College London, University of London; 2006.

Lahm SA. Ecology and economics of human/wildlife interaction in northeastern Gabon. Dissertation. New York University, New York,

Lupo K, Schmitt DN. Small prey hunting technology and zooarchaeological measures of taxonomic diversity and abundance: Ethnoarchaeological evidence from Central African forest foragers. J Anthro Archaeo 2005; 24: 335–353.

Mbete P, Ngokaka C, Akouango F, Bonazebi N, Vouidibio J. Evaluation of the depletion of game by hunting around the Park National of Odzala Kokoua and the impact on biodiversity degradation. J Anim Plant Sci 2010; 8: 1061-1069.

Muchaal PK, Ngandjui G. Impact of village hunting on wildlife populations in the western Dja Reserve, Cameroon. Conserv Biol 1999; 13: 385–396.

Noss AJ. Duikers, cables and nets: A cultural ecology of hunting in a Central African forest. Ph.D. thesis. University of Florida, Gainesville, FL, USA; 1995.

Noss AJ. The impact of cable snare hunting on wildlife population in the forests of the Central Africa. Conserv Biol 1998; 12: 390-398.

Noss AJ. Cable snares and nets in the Central African Republic. In: Robinson JG, Bennett EL, editors. Hunting for sustainability in tropical forests. Columbia University Press., New York; 2000. pp. 282–304.

Riddell M. Assessing the impacts of conservation and commercial forestry on livelihoods in northern Republic of Congo. Conserv Soc 2013; 11: 199-217.

Wilkie DS. Impact of swidden agriculture and subsistence hunting on diversity and abundance of exploited fauna in the Ituri forest of North-East Zaire, PhD Thesis, University of Massachusetts; 1987.

Willcox AS, Nambu DM. Wildlife hunting practices and bushmeat dynamics of the Banyangi and Mbo People of southwestern Cameroon. Biol Conserv 2007; 134: 251-261.

Yasuoka H. The sustainability of duiker (*Cephalophus* spp.) hunting for the Baka hunter-gatherers in southeastern Cameroon. African Study

Monographs Supplementary Issue 2006a; 33: 95–120.

Yasuoka H. Long-term foraging expedition (molongo) among the Baka hunter-gatherers in the northwestern Congo Basin, with special reference to the "wild yam question." 2006b; Hum Ecol 34: 275–296.

Yasuoka H. The variety of forest vegetations in southeastern Cameroon, with special reference to the availability of wild yams for the forest hunter-gatherers. Afr Study Monogr 2009; 30: 89-119.

Yasuoka H, Hirai M, Kamgaing TOW, Dzefack ZCB, Kamdoum ECB, Bobo KS. Changes in the composition of hunting catches in southeastern Cameroon: a promising approach for collaborative wildlife management between ecologists and local hunters. Ecol Soc 2015; 20(4):25. http://dx.doi.org/10.5751/ES-08041-200425
